# Rates of Compliance in South Indian American Communities of Southern California Regarding Cancer Screening

**DOI:** 10.3390/clinpract14010026

**Published:** 2024-02-08

**Authors:** Bhavana Seelam, Ria Sandhu, Mariam Alam, Akhila Kethireddy, Isain Zapata

**Affiliations:** 1 Rocky Vista University College of Osteopathic Medicine, Ivins, UT 84738, USA; 2 Rocky Vista University College of Osteopathic Medicine, Englewood, CO 80112, USA; 3 M. S. Ramaiah Medical College, Bangalore 560054, Karnataka, India; 4 Department of Biomedical Sciences, Rocky Vista University College of Osteopathic Medicine, Englewood, CO 80112, USA

**Keywords:** cancer screening, South Indian, colorectal, lung, mammography, pap smear

## Abstract

Background: Studies have shown lower rates of cancer screening and high mortality rates among all Asian Americans than among non-Hispanic White populations. However, most of these studies often confound diverse Asian American subgroups with limited data on cancer screening for Indian Americans, with this group being particularly interesting because of their counterintuitive socioeconomic status. For this reason, the objective of this study is to evaluate knowledge of the United States Preventive Services Task Force (USPSTF) cancer screening guidelines and compliance among South Indian Americans residing in Southern California. Methods: This was a cross-sectional study gathering community responses through an electronic survey. The survey reports knowledge of USPSTF screening guidelines and participant compliance rates. Rates were further compared to non-Hispanic White populations from official sources. Results: South Indian Americans residing in California had lower rates of compliance for colorectal, lung, and breast cancer screening when compared to that of non-Hispanic White populations in the same region, with the exception of cervical cancer screening rates. Conclusion: Understanding the cultural characteristics of special populations, such as Indian Americans, can help communities adhere to more effective screening practices that can improve outcomes.

## 1. Introduction

Asian Americans are the fastest-growing racial/ethnic group in the United States that is expected to pass 35 million by 2060 [1]. Within this group of Asian Americans, those immigrating from India (Indian Americans) represent the second largest group with 4.7 million following those immigrating from China [2]. Indian Americans are the highest-earning ethnic group in the United States, even above non-Hispanic White populations, and are more likely to pursue higher education and hold management positions. They have some of the lowest poverty rates and are less likely to be uninsured [2,3]. This perceived privileged position has granted them the label of “model minority” [4].

Despite these advantages, health-related outcomes in this population have lagged. A quite notable aspect of this discrepancy is malignant neoplasms, which are the leading cause of death among Indian Americans in the United States [5], and the second leading cause of death in California after cardiovascular disease [6]. This is perplexing considering that the incidence of cancers in Indian Americans is comparable to non-Asian-born populations [7] but is lower than non-Hispanic white populations [8]. When considering mortality rates, malignant neoplasm-related fatalities in non-Hispanic white populations have been steadily decreasing while Indian-American fatalities have not [8]. Despite having a lower genetic risk for some cancers [9], discrepancies in mortality rates suggest disparities associated with management or screening efficacy may exist that are not being addressed. These may exist despite the higher-than-average socioeconomic status of this ethnic group.

In the United States, the USPSTF (United States Preventive Services Task Force) prepares the recommended screening guidelines used by medical providers. These guidelines cover all ailments including cancers such as colorectal, lung, cervical, and breast cancer. Detailed guidelines are available at the official USPSTF site [10]. Asian Americans often display elevated mortality rates in comparison to other ethnicities because they are often diagnosed during the advanced stages of cancer [11,12]. This makes preventive care and cancer screening for this population an essential area to improve.

Therefore, the purpose of the study was to evaluate knowledge of USPSTF cancer screening guidelines and compliance among South Indian Americans residing in Southern California. The significance of the study resides in highlighting the importance of cultural considerations when assessing the cancer risk of very specific populations.

## 2. Materials and Methods

### 2.1. Participants

This is a cross-sectional study focused on South Asian communities currently residing in California; we specifically tailored our survey to Indian Americans who have cultural heritage from the states of Andhra Pradesh, Telangana, Karnataka, Tamil Nadu, and Kerala. We have also focused on the communities that are currently residing in Orange County and Los Angeles County, which have ~63,000 and ~111,000 Indian Americans, respectively, according to 2020 U.S. Census data [2]. Participants had to have a basic level of English reading and comprehension skills to participate in the survey. All participants received an informed consent form to participate in the study voluntarily. This study was vetted by The Rocky Vista University Institutional Review Board and approved as exempt (IRB#2022-175).

### 2.2. Survey and Survey Distribution

The survey was administered online using Qualtrics XM Platform (Qualtrics International Inc., Provo, UT). The online survey required approximately 3 min to complete. The questionnaire consisted of a few questions based on the gender of the participant. All participants were asked if they knew the United States Preventive Services Task Force (USPSTF) guidelines, followed by questions asked to assess adherence regarding the guidelines on colorectal, lung, cervical, and breast cancer screening. The USPSTF is an independent panel of physicians and scientific experts that develops recommendations for clinical preventive practices based on evidence of effectiveness. The panel is assembled by volunteers and is funded by the Agency of Healthcare Research and Quality of the U.S. Department of Health and Human Services. Detailed guidelines are presented at the USPSTF official site.

Men were asked their age group, colorectal cancer screening adherence, and lung cancer screening adherence. Multiple screening options such as fecal immunochemical test (FIT), fecal occult blood test (FOBT), colonoscopy, and sigmoidoscopy were presented as options that would display adherence to colorectal cancer screening. The lung cancer screening question was solely an option for those who smoked more than or equal to 20 cigarettes per day. Those who did not smoke were given the option to answer as such. Women were asked the same as men with the addition of questions about their adherence to pap smears and mammograms. Those below the age of 45 were given the option to bypass these questions. On review, the age grouping options in this study did not allow participants who were below the age required for colonoscopy to respond that they were below the age required. For them, the only other option therefore was to choose that the colonoscopy was contraindicated. This issue was accounted for in the data. Survey questions are presented in Table 1.

Screening adherence questions were simplified for survey participants’ convenience. Adherence to screening was based on Grade A and B guidelines [10]. Guidelines below these grade lines were not considered in our study. First-degree relatives and hereditary risk factors were also not considered when querying participants about compliance.

The survey was distributed through targeted social media on WhatsApp (Meta Platforms, Inc., Menlo Park, CA, USA), through community emails, and via word of mouth among members of the community from December 2022 to January 2023. Participants were provided survey links and QR codes through which they could access the survey on their mobile or desktop.

### 2.3. Data Analysis

Survey data were exported from the Qualtrics XM Platform and evaluated descriptively (frequencies and percentages) using SAS/STAT v.9.14 (SAS Institute Inc., Cary, NC, USA). Since all questions were categorical, associations were evaluated using contingency tables using Exact Chi-square tests in PROC FREQ. Significant associations were declared at *p* ≤ 0.05. We make a direct comparison of compliance rates to non-Hispanic White values from the CDC [13] and a recent independent study (cited in the text) to provide additional context.

## 3. Results

### 3.1. Screening Rates in the South Indian American Population Surveyed

The purpose of this study was to evaluate frequencies and associations of knowledge of USPSTF guidelines and compliance for cancer screening in participants of South Indian heritage living in Southern California. A total of 164 South Indian Americans residing in Southern California participated in this study. Out of this population, 93 (56.7%) were male and 71 (43.3%) were female. The survey did not establish the origin state of the survey taker and was focused on encompassing all South Indian communities without distinction. Adult South Indian Americans aged 21 or older were eligible to be participants in the study. Frequency data are presented in Table 2.

There were no significant differences in people reporting knowing USPSTF guidelines by sex (Exact *p* = 0.8227); however, there were differences by age group (Exact *p* = 0.0276) where participants over 45 years were more likely to know about guidelines (OR = 3.86). Participants who reported having colorectal cancer screenings were more aware of USPSTF guidelines (Exact *p* = 0.0026, OR = 1.54). Similarly, female participants who reported not having a mammogram were more likely to belong to the 45-year-old or younger group (*p* = 0.0210, OR = 1.40). No other associations were observed.

### 3.2. Comparison to Non-Hispanic White Population Screening Rates

The CDC reported colorectal cancer screening rates for non-Hispanic White populations in 2019, which were 73.5% nationally, 66.2% in California [13], and 83.1% in an independent national population study [14], which is considerably higher than the 33.6% observed in the participants in this South Indian American sample. The discrepancy is slightly lower for participants over 45 years of age (39.4%) but still below half for non-Hispanic Whites. Lung cancer screening rates for the non-Hispanic White population in an independent population-based 2020 sample was 17.5% [15], which is over 3 times larger than the 5.3% that was observed in the sample in this study. The Centers for Disease Control (CDC) does not compile data for lung cancer screening. The CDC reported cervical cancer screening rates for non-Hispanic White female populations in 2019, which were 78.9% nationally, 84.5% in California [13], and 57.7% in an independent study using 2019 population-based data [16]. These rates are lower than observed in this study for females over 45 years of age, at 81.0%. CDC reported mammograms for breast cancer screening rates for the non-Hispanic White female population in 2019 was 77.6% nationally, 77.3% in California [13] or 79.6% in an independent study using 2018 data [17], while it was 55.6% in females over 45 years of age in this sample. Summarized CDC data are presented in Table 3.

## 4. Discussion

Overall, USPSTF guideline knowledge is low in the population sampled; this becomes relevant when considering low compliance rates in comparison to the non-Hispanic White population cancer screening rates, with the exception of cervical cancer screenings. Our findings indicate a large gap in knowledge that may be responsible for low screening compliance. This gap may originate from poor educational practices and screening within the natal South Indian population before immigrating to the United States. In India, cancer screening guidelines are non-existent for colorectal cancer [18] and lung cancer [19], with screening being performed for the most part at tertiary referral hospital centers [18,19]. For females, breast cancer screening ranges from scattered [20] to non-existent [21]. A study evaluating cervical cancer screening knowledge and practice detected a significant proportion of participants with a positive attitude towards screening, at 43.64%, although only 13.22% actually obtained them [22]. All these statistics indicate a large gap in screening practice.

Several studies have proposed culturally appropriate educational strategies for cancer screening [23] that consider a multitude of aspects that include disparities. However, since the Indian American population is not traditionally economically disadvantaged [2,3] and is often seen as a privileged “model minority” [4], many of the approaches designed to address minorities that assume some degree of poverty are ineffective in this population. A study evaluating the socioeconomic advantages of Indian Americans in the region of Houston, Texas [24], detected the exact same deficiencies as in our study where higher socioeconomic status in this population was associated with high cervical cancer screening rates but not breast, prostate, and colon cancer screening.

Other factors such as racism or anti-Asian rhetoric may contribute to the low education and compliance rates [12,25]. Even though this is a difficult systemic issue to resolve, efforts are being made in the mainstream [26] and at an institutional level to combat this sentiment [27].

The study presented is limited by its sample size representation, even when it validates the findings of other studies [24]. The nature of this study is based on self-reported answers that may introduce some noise into the data. In this study, there were additional limitations in data completeness where a portion of participants chose not to disclose their age. Further studies should be extended to diverse geographic regions and should consider additional effects such as ethnic and cultural admixture along with lifestyle changes. Further confirmation of screening rates and practices is also recommended to avoid the possibility of discrepancies due to answers being self-reported. These studies should also incorporate the rationale and personal experiences of the community and how these could be improved to increase preventive care.

## 5. Conclusions

In summary, this study presents further evidence of associations and characteristics about the Indian American population and their cancer screening practices. Studies like this will be important for future research on implementing an educational approach for teaching USPSTF guidelines targeting at-risk groups. Moreover, incorporating the feedback of special populations such as the one presented in this study can aid in refining UPSTF guidelines to include considerations related to cultural beliefs and barriers to screening.

## Figures and Tables

**Table 1:** Survey questions. The survey was implemented in Qualtrics and distributed digitally through a QR code to people meeting the inclusion criteria.

Question	Answer Options
1. Are you a South Indian currently residing in the state of California? (Telugu, Tamil, Kannada, Malayalam)	Yes/No (A yes is required to be eligible)
2. Do you know what the USPSTF guidelines for screening are?	Yes/No
3. What sex were you assigned at birth?	Male/Female
4. What is your age?	Under 45 years old/45 years old or above
5. After turning 45 years old, have you been getting a FIT or FOBT test every year OR flexible sigmoidoscopy/CT colon every 5 years OR a colonoscopy every 10 years OR a sigmoidoscopy every 5 years? (FIT = Fecal Immunochemical Test; FOBT = Fecal Occult Blood Test; CT = Computerized Tomography)	Yes/No/These tests are contraindicated for me
6. If you are between ages 50–80 and currently smoke more than or equal to 1 pack/day for more than 20 years, have you ever gotten a low-dose chest CT scan?	Yes/No/I do not smoke/I am below age 50
7. If you are a female above age 21, have you gotten your pap smear every 3 years; or if above age 29, a pap w/HPV testing every 5 years until age 65?	Yes/No/I am below 21
8. If you are above age 50, have you had a mammogram?	Yes, every 2 years after turning 50/Yes, irregularly/I’ve had a mastectomy/I am below 50/No

**Table 2:** Descriptive statistics of the survey participants. All participants asserted being of South Indian heritage living in the Southern California region. Contraindicated responses marked with an asterisk (*) are for those persons who are recognized as under the recommended age.

Variable	Full Dataset		Females		Males		45 Years or Younger		Over 45 Years	
	Frequency	%	Frequency	%	Frequency	%	Frequency	%	Frequency	%
Says they know USPSTF guidelines										
No	142	86.6	62	87.3	80	86.0	49	92.5	54	76.1
Yes	22	13.4	9	12.7	13	14.0	4	7.6	17	23.9
Sex										
Female	71	43.3	-	-	-	-	26	49.1	21	29.6
Male	93	56.7	-	-	-	-	27	50.9	50	70.4
Age										
45 years or younger	53	42.7	26	55.3	27	35.1	-	-	-	-
Over 45 years	71	57.3	21	44.7	50	64.9	-	-	-	-
Colorectal cancer screening										
No	75	63.0	25	61.0	50	64.1	28	71.8	42	59.2
Yes	40	33.6	14	34.2	26	33.3	9	23.1	28	39.4
Contraindicated *	4	3.4	2	4.9	2	2.6	2	5.13	1	1.4
Lung cancer screening (Chest CT Scan)										
No	18	94.7	5	100.0	13	92.9	4	80.0	13	100.0
Yes	1	5.3	0	0.0	1	7.1	1	20.0	0	0.0
Cervical cancer screening (Pap smear)										
No	10	16.7	10	16.7	-	-	4	15.4	4	19.1
Yes	50	83.3	50	83.3	-	-	22	84.6	17	81.0
Breast cancer screening (Mammogram)										
No	7	38.9	7	38.9	-	-	5	100.0	2	22.2
Yes	7	38.9	7	38.9	-	-	0	0.0	5	55.6
Irregularly	4	22.2	4	22.2	-	-	0	0.0	2	22.2

**Table 3:** Compliance rates (%) for colorectal, cervical, and breast cancer screenings from CDC data and this study. National-level and state-level data are available by race while county-level data are not.

Cancer Type Screening	CDC Data						
	National (NHW)	California (NHW)	Los Angeles County (All Races)	Orange County (All Races)	San Bernardino County (All Races)	Riverside County (All Races)	This Study
Colorectal cancer screening (both sexes)	73.5	66.2	58.1	61.9	57.4	60.6	33.6
Cervical cancer screening (females)	78.9	84.5	80.8	81.8	81	81.9	81
Breast cancer screening (females)	77.6	77.3	70.9	67.7	71.9	69.3	55.6

## Data Availability

The datasets generated during and/or analyzed during the current study are available from the corresponding author upon reasonable request.
